# Estimating hypoxia-induced brain dysfunction and cognitive decline through exhaled breath monitoring

**DOI:** 10.1186/s12931-025-03296-5

**Published:** 2025-06-13

**Authors:** Sean W. Harshman, Kiersten J. Weatherbie, Alena R. Veigl, Anne E. Jung, Madison A. Stoner-Dixon, Aubrianne I. Dash, Christopher J. Land, Dylan T. Slizewski, Eli F. Kelley, Jennifer Schwanekamp-Kerr, Timothy Halverson, Christina N. Davidson, Christopher W. Myers, Kara J. Blacker, Jennifer A. Martin, Rhonda L. Pitsch

**Affiliations:** 1 711th Human Performance Wing, Air Force Research Lab, 2510 Fifth Street, Building 840, Area B, Wright-Patterson AFB, 45433 OH USA; 2 Naval Medical Research Unit Dayton, 2624 Q Street, Building 851, Area B, Wright-Patterson AFB, OH USA; 3 Leidos Inc, Naval Medical Research Unit Dayton, 2624 Q Street, Building 851, Area B, Wright-Patterson AFB, OH USA; 4 UES Inc., 711th Human Performance Wing, Air Force Research Lab, 2510 Fifth Street, Building 840, Area B, Wright-Patterson AFB, OH USA; 5 Aptima, Inc, 2555 University Blvd, Suite 300, Fairborn, OH USA

## Abstract

**Background:**

Hypoxia remains a concern for aircrew operating high performance aircraft. Sensing and mitigating hypoxia is a line of active research within the US Air Force and US Navy. It is hypothesized that changes in exhaled breath volatile organic compound content could indicate, not only changes in oxygen saturation (SpO_2_), but also brain activity and cognitive function.

**Methods:**

On-line exhaled breath monitoring via proton transfer reaction mass spectrometry was used to observe changes in volatile organic compound concentrations during mask-free hypoxic exposures. Additionally, electroencephalography measurements in response to an odd-ball paradigm and cognitive tasks were collected throughout the exposures.

**Results:**

The data show hypoxic exposures induced a physiological response including a significant reduction in SpO_2_, a decrease in the electroencephalography waveform peak-to-peak amplitude (*p* < 0.05), a significant increase in psychomotor vigilance test response time, and an increase in perceived symptomatology. Exhaled breath results indicate 19 volatile organic compound features are significantly different between hypoxia and normoxia (*p* < 0.05) with 13 showing an increase in exhaled breath compared to background measurements (*p* < 0.05). Linear mixed modeling with stepwise reduction demonstrates 7 of the features are significantly indicative of changes in SpO_2_ with 3 and 4 features indicative of changes in brain wave functions and psychomotor vigilance test response times, respectively.

**Conclusions:**

The data establish, for the first time, differences in exhaled breath volatile concentrations that indicate changes in cognition derived from hypoxic insult.

**Supplementary Information:**

The online version contains supplementary material available at 10.1186/s12931-025-03296-5.

## Introduction

Hypoxia, or low level of oxygen within the tissues, is a well-known risk in the tactical aviation community. Commonly encountered at high altitudes, acute hypoxic exposure negatively impacts operational procedures such as maintaining altitude, constant airspeed, and directional heading [1–4]. The reduction in performance is plausibly derived from hypoxia’s adverse effect on a range of neurophysiological systems including cognition, sensory perception, and motor control, resulting in a wide variety of subjective, self-reported symptoms [5–7]. As part of their training, military aviators engage in controlled hypoxic exposures to recognize their symptoms; however, there is wide-ranging variability both between and within individuals [8]. While individual perception has been utilized by the aviation community for decades, alternative means for hypoxia monitoring, without direct impact on job duties, are necessary to protect the safety of aviators in flight. It is hypothesized that monitoring changes in exhaled breath volatile organic compound (VOC) abundances could fulfill this need.

Exhaled breath has been evaluated since the early 1970s with changes in VOC signatures connected to many physiological conditions and disease states [9, 10]. Of interest, previous work by our group investigated the use of exhaled breath VOCs to predict the onset of acute arterial hypoxemia using a mask-based Reduced Oxygen Breathing Device (ROBD) to provide 25,000ft O_2_ equivalent exposures via a flight mask [11, 12]. These studies established the plausibility for the prediction of hypoxemia, measured by a reduction in peripheral oxygen saturation (SpO_2_), using exhaled breath VOC abundances. However, flight masks utilize on-demand breathing and frequently require labored or forced breathing, no matter the O_2_ concentration provided. Therefore, while proper controls were used, changes in exhaled VOCs within these studies may be related to labored breathing rather than hypoxemia itself. As a result, an alternative means to conduct hypoxia exposures independent of a flight mask was required to fully identify volatile changes in exhaled breath. As a solution, the Reduced Oxygen Breathing Environment (ROBE), a plexiglass chamber where the O_2_ content of the room is regulated, was developed to afford exposing individuals to changes in O_2_ content independent of a flight mask.

While controlled hypoxic exposures within the ROBE have been shown to induce a significant decrease in an individual’s peripheral oxygen saturation (SpO_2_), the impact of lowered SpO_2_ on brain function and cognition is the primary operational endpoint [11, 12]. Data have shown that acute hypoxia exposures illustrate a significant reduction in electroencephalography (EEG) waveform amplitudes compared to normoxic conditions [7, 13]. Furthermore, participants undergoing hypoxic exposure have a reduction in cognitive performance relating to response time latency, impaired working memory, and altered decision making [5, 6, 14–21]. Therefore, linking exhaled breath VOC changes to hypoxia-induced disruption to brain activity and cognitive performance is of extreme interest to enhance aviators’ safety.

## Experimental

### Participants

**Fig. 1:**
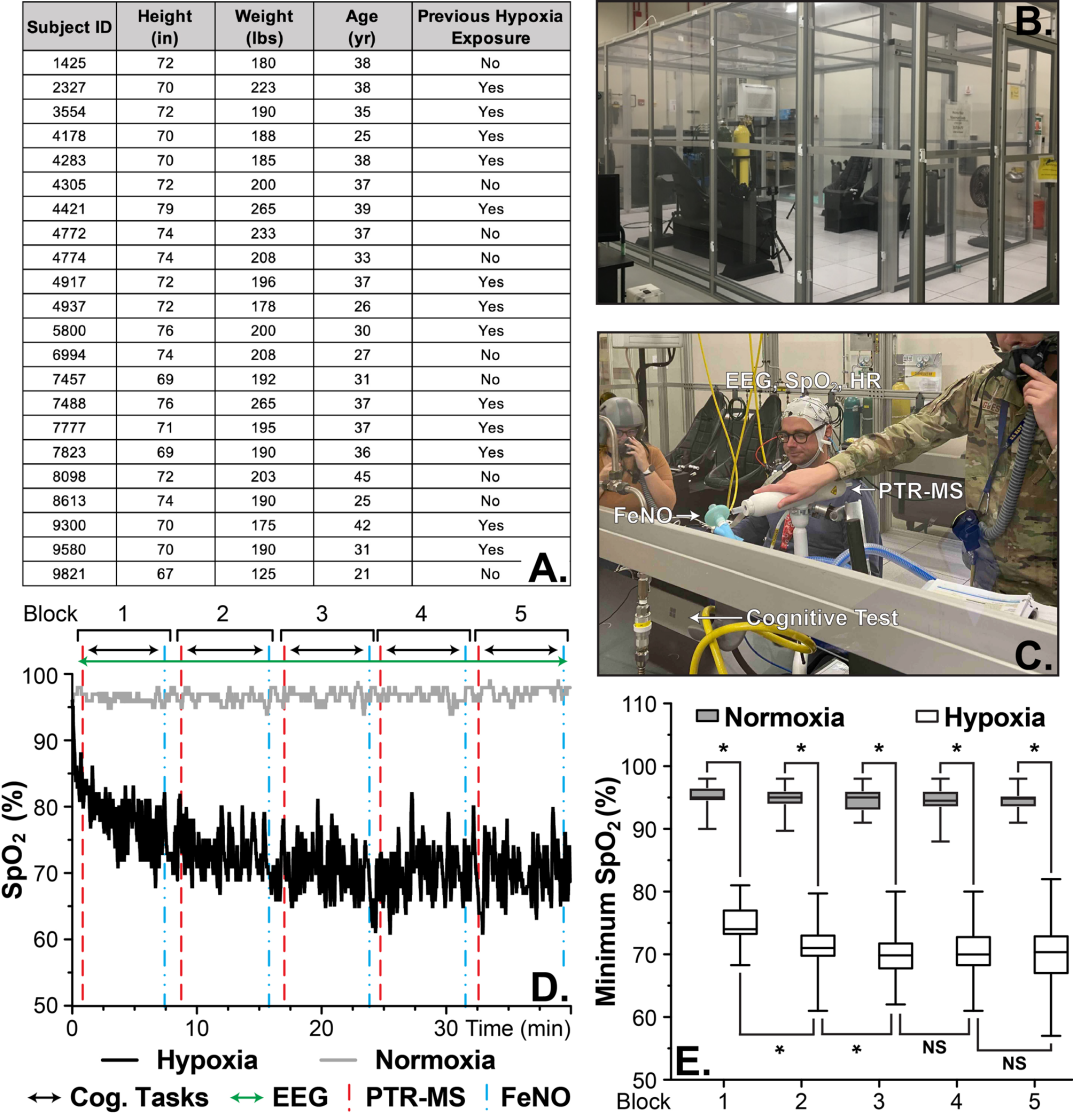
Experimental setup and measured oxygen saturation for each exposure, normoxia and hypoxia. **(A)** A summary of the available demographic data. **(B)** An image of the Reduced Oxygen Breathing Environment (ROBE) used for the two exposures. **(C)** An image of the experimental setup within the ROBE. **(D)** A figure depicting both the sampling times and a representative SpO_2_ trace from normoxic and hypoxic exposures. **(E)** A box plot of the minimum SpO_2_ from the exposures by sampling block. * indicate *p* < 0.05, NS = not significant, and error bars represent the min/max values. The results show a significant reduction in oxygen saturation (SpO_2_) in the hypoxic condition compared to the normoxic exposure

A total of 31 male adults enrolled in the study with 22 participants completing all aspects of the protocol. Please refer to Fig. 1A for demographic information. Participants self-reported normal or corrected-to-normal vision; normal hearing; no history of psychological, neurological, or other medical diagnoses; no use of tobacco in the past six months; and no excessive alcohol use. Thirteen participants reported previously experiencing hypoxia. The study protocol was approved by the Naval Medical Research Unit– Dayton’s (NAMRU-D) Institutional Review Board (NAMRUD.2022.0003) in accordance with the principles embodied in the Declaration of Helsinki and local statutory requirements and all participants provided written informed consent prior to participation.

### Hypoxia & normoxia exposures

The exposures were single-blinded, with participants performing three visits on separate days. The initial visit, participants provided written informed consent and demographic information; completed screenings for eligibility; and conducted a familiarization session for each cognitive task. Approximately one week following the initial visit, participants completed two experimental visits at approximately the same start time in counterbalanced order of exposure condition, altitude oxygen (hypoxia) and sea level oxygen (normoxia), with at least 36 h between the experimental visits (µ = 4.7 ± 5.90 days).

Each 45-minute exposure was conducted in a ROBE (Fig. 1B), a normobaric hypoxia chamber, with the ROBE held at approximately 10.6% O_2_ (17,500ft equivalent) for the hypoxic condition and 21% O_2_ for the normoxic condition. Please refer to Supplemental Data 1 for a representative example and a box plot of the measured O_2_ of the ROBE during the exposures. Prior to entering the ROBE, an EEG cap was donned, a fractional exhaled nitric oxide (FeNO) sample was collected, and participants provided blood samples. Upon entering the ROBE, participants immediately began tasks, which consisted of five, 8-minute testing blocks where participants provided two breath samples within the PTR-MS, performed six minutes of randomized cognitive tasks, and provided a FeNO sample (Fig. 1C and D). Please refer to future sections for details of each task within the ROBE. Upon completion of the exposure, participants were provided 21% O_2_ through a Hans Rudolph oronasal mask until their peripheral oxygen saturation (SpO_2_) was ≥ 90%. Participants exited the ROBE, completed a hypoxia symptom questionnaire (HSQ), and provided an additional FeNO sample as well as a blood sample and an oral/salivary sample. Of note, while the blood (global metabolomics) and salivary samples were collected within this study, they will not be discussed here.

### Physiological measurements

Throughout both exposures, participants’ SpO_2_ and heart rate (HR) were monitored and recorded at 1 Hz. Both measures were acquired via a finger-mounted pulse oximeter and recorded by an Apple iPad via Bluetooth connection (Nonin Medical Inc., Plymouth, MN, USA). Please refer to Fig. 1D and E and Supplemental Data 2 for representative measured SpO_2_ and HR data along with summary data for all participants. The three lowest SpO_2_ (minSpO_2_) values within a block were averaged and used for further analysis. A safety cut-off criterion of 60% SpO_2_ for all participants was used, whereby an individual was provided supplemental oxygen and their exposure prematurely ended upon exceeding this limit.

### Phlebotomy

Approximately, 16mL total of unfasted whole blood was drawn from each volunteer’s arm (4mL into each K2 EDTA (purple top), Lithium Heparin (green top), CAT serum (red top), and PAXgene RNA tubes (BD Biosciences, Franklin Lakes, NJ, USA, & Greiner Bio-One Monroe, NC, USA). The collection area was cleansed with a 70% alcohol swab and wiped with sterile gauze (BD Biosciences). A Greiner Bio-one 21G x 1” multi drawing needle with hub was inserted into the vein and tubes were inserted onto the needle assembly until full. The needle assembly was removed, and sterile gauze was placed over the site. Each sample type was prepared as instructed by the manufacturer, aliquoted, and stored at -80 °C until analyzed.

### iSTAT blood chemistry & gases

Blood chemistry and gases were evaluated using an i-STAT point of care device affixed with an i-STAT CG8 + cartridge which measures sodium; potassium; ionized calcium; glucose; hematocrit; hemoglobin; pH; partial pressure of carbon dioxide (PCO_2_); partial pressure of oxygen (PO_2_); total carbon dioxide (TCO_2_); bicarbonate (HCO_3_); base excess (BE); and oxygen saturation (sO_2_) (Abbott Laboratories, Abbott Park, IL, USA). Briefly, the iSTAT instrument was evaluated for quality control and the CG8 + cartridge was warmed to room temperature. A small portion (~ 100µL) of sample was removed from a thoroughly mixed lithium heparin (green top) blood tube within 10 min of sampling and added to the device. Data was recorded and tabulated manually.

### Hypoxia symptom questionnaire (HSQ)

A 15-item hypoxia symptom questionnaire (HSQ) was utilized to measure each participants’ symptomology following each exposure on a 4-point scale (0 = not observed, 3 = severe) [22]. The HSQ was developed directly from the U.S. Navy’s hypoxia familiarization training program and contains items related to commonly experienced hypoxia symptoms.

### Cognitive tasks

Participants were seated approximately 65 cm from a 13.5” tablet affixed with a standard keyboard modified to cover the symbols and reveal only the numbers (Fig. 1C, HP, Palo Alto, CA, USA, ). Each task, detailed below, was presented randomly for two-minutes within each six-minute block, on the tablet screen. All cognitive tasks were custom built in NetBeans and were run using Apache Ant 1.8.0.

### Psychomotor vigilance task

The Psychomotor Vigilance Task (PVT) is a simple visual reaction time (RT) test. Briefly, participants were presented with a red counter on a grey background which counted up from zero. Participants were instructed to strike any key on the keyboard once the number was observed. RT was the time, in milliseconds, from stimuli appearance on the screen to key striking. The stimuli remained on-screen for 1,000ms after a response was recorded to provide the participant with performance feedback. The interstimulus intervals were distributed randomly from 1,000ms to 5,000ms [23]. RTs were filtered, tabulated, and recorded.

### Digit symbol substitution task

The Digit Symbol Substitution Task (DSST) is a measure of associative declarative memory. First, participants were presented with a sequence of ten pairings of numbers and symbols on the keyboard (e.g., 1 and!, 2 and @, 3 and #, 4 and $, and so on). The first presentation of a number was paired with the corresponding symbol. Once all ten pairings had been introduced, only symbols (e.g.,!) were presented on-screen in a random fashion. Participants were instructed to press the number on the keyboard that corresponded with the presented symbol stimulus. Stimuli were presented in a random order for 5,000ms, with 1,000ms between stimulus presentations. Error in associations and response times were tabulated and recorded [24].

### Change signal task

The Change Signal Task (CST) measures an individual’s response to inhibition. Participants were initially presented with a “Go Signal” displayed on-screen (i.e., an arrow pointing left or right) and instructed to indicate the direction of the “Go Signal” by striking the corresponding arrow key on the keyboard. In 33% of trials, a “Change Signal” (a larger arrow) was presented after the “Go Signal”, indicating to participants to respond on the keyboard by striking the arrow key pointing in the opposite direction of the “Go Signal”. The time delay between the “Go Signal” and the “Change Signal” (stimulus onset asynchrony) fluctuated between 20ms and 800ms based on a staircase procedure, such that the initial delay was 200ms and decreased by 50ms when participants successfully changed responses and increased by 100ms when participants unsuccessfully changed response. Two error conditions were presented (low and high), which differed with respect to the color of the “Change Signal’s” arrow [25]. Error in “Change Signal” accuracy and reaction time were tabulated and recorded.

### EEG: Event-Related potentials (ERP)

The EEG data were recorded continuously from 32 electrodes in an elastic electrode cap uniformly covering the entire scalp, at 500 Hz with electrode impedance for all channels < 25kΩ and referenced to FCz in DC mode (LiveAmp, Brain Products GmbH, Gilching, Germany). Auditory tones were presented to the participant every 500ms at 85dB sound pressure level throughout the exposure within the ROBE via Etymotic ER3-A insert earphones (Lucid Hearing, Fort Worth, TX, USA). Participants were instructed to ignore the tones and to focus only on the tasks within the blocks. A passive auditory oddball paradigm was used that comprised a sequence of tones (*n* = 5,100). 85% of the tones were considered standard (50ms at 1,000 Hz) and 15% were deviants, either differing in duration (100ms at 1000 Hz) or both duration and frequency (i.e., “double-deviant”; 100ms at 1,100 Hz) [26–30]. All tones had a 5ms rise/fall.

The EEG data were processed using the Fieldtrip software package within the MATLAB operating environment [31]. The data were segmented into epochs covering the time from 100ms prior to and 500ms following the onset of each auditory stimulus presentation. The data were low-pass filtered at 20 Hz and referenced to the average of both mastoids (TP9, TP10). An independent components analysis (ICA) was performed and the eye blink and lateral eye movement components were removed. The EEG waveforms from frontal electrodes (i.e., Fp1, Fp2) were visually inspected for voltage fluctuations typical of gross motor movements (i.e., amplitude > ± 75µV) and trials containing these types of artifacts were rejected. The average waveforms were calculated for deviant and standard stimulus response within the six-minute cognitive task blocks throughout each exposure, using electrode Fz for analyses. Of note, one participant had excessive Fz noise and alternatively the Cz electrode was used. In this case, the nearest midline electrode to Fz was chosen based on visual inspection of topographic maps rather than a group of adjacent lateral electrodes. These data show the MMN/P3a complex was maximal over the frontal midline sites and similarly distributed between Fz and Cz [7].

The difference waves were calculated for each six-minute cognitive task block by subtracting the averaged standard response from the averaged deviant response. Additionally, the difference waves were calculated for the entire exposure to examine exposure changes. The mismatch negativity (MMN) was defined as the most negative-going difference waveform between 50ms and 300ms and the P3a was defined as the most positive-going difference waveform between 150ms and 400ms. To compromise between peak- and mean-based measures, the mean amplitude in a 100ms window, centered around the peak such that the window varies for each dataset, was reported [32]. The MMN/P3a mean amplitude and latency as well as peak-to-peak amplitude (P2P) were found separately for each six-minute block of each visit.

### Proton transfer Reaction-Mass spectrometry (PTR-MS)

Upon entering the ROBE, participants situated themselves at the table as depicted in Fig. 1C. Within two minutes of entering, each participant provided duplicate complete exhalations, both upper and lower/tidal breath, approximately 30s apart into the Buffered End-Tidal Breathing (BET) inlet affixed with a new non-rebreathing mouthpiece. Thirty seconds was found to be the approximate time that the breath signal within the instrument returned to baseline following an exhalation. Breath was sampled from the BET via a 70 °C heated line into an Ionicon Analytik Proton Transfer Reaction Time of Flight Mass Spectrometer 4000 sampling at approximately 50mL min^-1^ (PTR-MS, Innsbruck, Austria). All exhaled breath data were collected with H3O^+^ ionization utilizing the instrumental parameters described previously [33]. Briefly, spectra were acquired every 250ms over 5-389 m/z with a max flight time of 25µs and a 2µs extraction time. The source was operated with a drift temperature of 70 °C, a drift pressure of 2.8mbar, and a drift voltage of 680 V yielding an approximate E/N ratio of 124Td. The time of flight (TOF) detector was operated at approximately 4 × 10^− 7^ mbar. Every 20s during acquisition, the mass axis was automatically recalibrated using the H_3_O^+^ O^18^ isotope (21.02 m/z) and two PerMaSCal fragments (203.95 m/z and 330.85 m/z). The instrument was run continuously throughout the entire exposure. Daily checks of 100ppb isoprene dry compressed gas standard were used to verify instrument performance (≤ 10% RSD across all data collection days) and approximately 20 background spectra within the ROBE were collected prior to each exposure using the settings described above. Hydronium (21.02 m/z) was monitored throughout the experiment and a representative example trace is provided in Supplemental Data 3.

All spectra were manually evaluated within Ionicon Analytik’s PTR-MS Viewer Software, as described previously (v. 3.4.4) [33]. Briefly, a Gauss fit function and a 3-point mass calibration was applied to the imported.h5 data files using ions 21.02 m/z, 203.95 m/z, and 330.85 m/z. Exhalations were visualized using acetone (59.052 m/z) and isoprene (69.075 m/z) ions within the “Analyze Trace” feature of the PTR-MS Viewer software. The parts-per-billion (ppb) values of ten scans within each tidal portion of the exhalation were exported for 135 total ions (features). The exported feature concentrations were averaged for the two exhalations within each block. The data were filtered to remove features related to hydronium and water adducts, those above m/z 200, and those below the instrument limit of detection (LOD) based on three standard deviations (3σ) of the zero-air measurement for each feature within exhaled breath samples. The remaining feature list, of 89 features, was exported for further statistical analysis as described in a future section. Please see Supplemental Data 4 for feature quantities and LOD values.

### Fractional exhaled nitric oxide (FeNO)

Fractional exhaled nitric oxide (FeNO) was measured following the cognitive testing within all blocks as well as pre/post-exposure on an EcoMedics Analyzer CLD88sp affixed with a DENOX 88 nitric oxide (NO) free air supply (Fig. 1C, ECO MEDICS, Switzerland). Briefly, the instrument was NO zero- and flow-calibrated on every test day with a span calibration monthly, as per the manufacturer’s recommendation. The participants were instructed to breath normally and gently in and out of the mouthpiece for 45s while utilizing a nose clip. The instrument was operated in “multiple breath NO test” mode. All data were manually evaluated in the EcoMedics Spiroware software (v. 3.3.2) as recommended by the manufacturer. In short, the plateau borders from each breath within the multiple breath test were reset using the software’s default, 60–80% of the exhaled volume. In the event the reset caused the plateau borders to be located near a portion of the breath that had a significant slope, the boundaries were manually adjusted while staying within 40–90% of the exhaled volume. The average FeNO and the coefficient of variance (%CV) for each breath within a sample was determined, by the software, and evaluated. The minimum and maximum values from each overall sample were removed until a %CV of > 5% for < 4 breaths or > 10% for > 5 breaths was achieved. The data was exported and further filtered to remove any breath with a Plateau Average Flow (mL s^-1^) less than 200mL s^-1^.

### Statistical analysis

Basic figures and statistics were created in PrismGraphpad (v. 10.1.1). All data cleaning and statistical analyses were performed in the RStudio software suite (v.2022.12.0 + 353). All p-values for testing among breath conditions and background were computed using a Wilcoxon signed rank test due to small sample size and lack of normality in the data. Statistical significance was defined as *p* < 0.05. The significant features were used to predict the average SpO_2_ through linear mixed modeling with data reduction as shown below. Additionally, application of the reduced model to EEG and PVT data was conducted as illustrated below.

The full linear mixed model is represented below, where *n* is 13 features, epsilon (ε) is the random error, the breath features (BF) are the fixed effects, and the participant is the random effect to account for non-independence of the repeated time point of each participant. The full model was evaluated with relation to SpO_2_ as shown in (A) below. The model was reduced by bidirectional reduction, yielding a reduced model consisting of a *k* of 8 features and epsilon (ε) is the random error (B below).


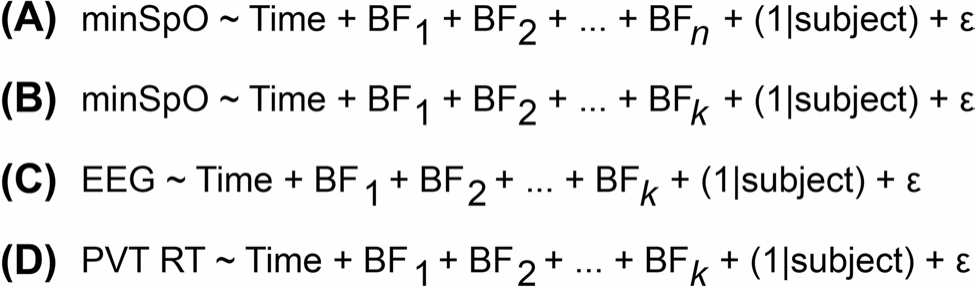


The reduced model was applied to the 8 features (*k*) and epsilon (ε) representing the random error with relation to changes in EEG amplitude and PVT reaction time as shown in (C) and (D) above.

## Results

### Hypoxia exposures

Twenty-two male participants with a median age of 37.0 ± 6.1 years, a median weight of 87.5 ± 13.2 kg, and a median height of 182.9 ± 7.1 cm completed all visits and therefore were used for analysis (Fig. 1A). To define if participants illustrated a physiological response from the exposures, SpO_2_ was continuously monitored. Figure 1D shows a representative time course of the measured SpO_2_ from an individual participant during the two exposures. Furthermore, Supplemental Data 2 A shows a boxplot of the overall measured minimum SpO_2_ (*p* < 0.0001) while Fig. 1E illustrates the minSpO_2_ by block for all participants (*p* < 0.05). Interestingly, the minSpO_2_ between Blocks 3–4, and 4–5 do not display a significant difference (*p* > 0.05) among these hypoxia blocks, suggesting a steady state level of SpO_2_ was achieved by Block 3 (Fig. 1E) [34, 35]. Collectively, the data show that the hypoxic exposures induced a significant reduction in individuals’ minSpO_2_ when compared to the normoxic exposures.

In addition to SpO_2_, HR was constantly monitored throughout each exposure. Supplemental Data 2B shows a representative time course of the measured HR among both exposures. Furthermore, Supplemental Data 2 C displays a boxplot of the overall maxHRs where a significant increase was observed (*p* < 0.0001) in the hypoxic exposure compared to the normoxic exposure. Finally, Supplemental Data 2D depicts a significant difference (*p* < 0.05) in the maxHR across all testing blocks. Overall, these data indicate a significant reduction in the measured oxygen saturation and significantly variable effects on heart rate of individuals undergoing altitude exposures.

Prior to and following both exposures, participants had their blood chemistry evaluated by an iSTAT point of care device. Supplemental Data 5 provides a summary of these results. While the samples were taken following recovery gas, the data show a significant difference in the delta (post-pre) between normoxia and hypoxia for several physiological processes (Supplemental Data 5). For instance, the hematocrit (red blood cells in relation to blood volume) and hemoglobin (protein responsible for oxygen delivery) are both statistically increased (*p* < 0.05) in the hypoxia condition suggesting a response by the body to attempt to increase oxygen flow and delivery. Furthermore, the hypoxic condition showed a significant (*p* < 0.05) increase in the blood glucose, indicating a potential impact on energy metabolism (Supplemental Data 5). These results are likely driven by the large variability in the normoxic condition’s delta values while the hypoxic condition shows very little change. Taken together, these data suggest significant changes in the blood composition in response to hypoxia exposure.

**Fig. 2:**
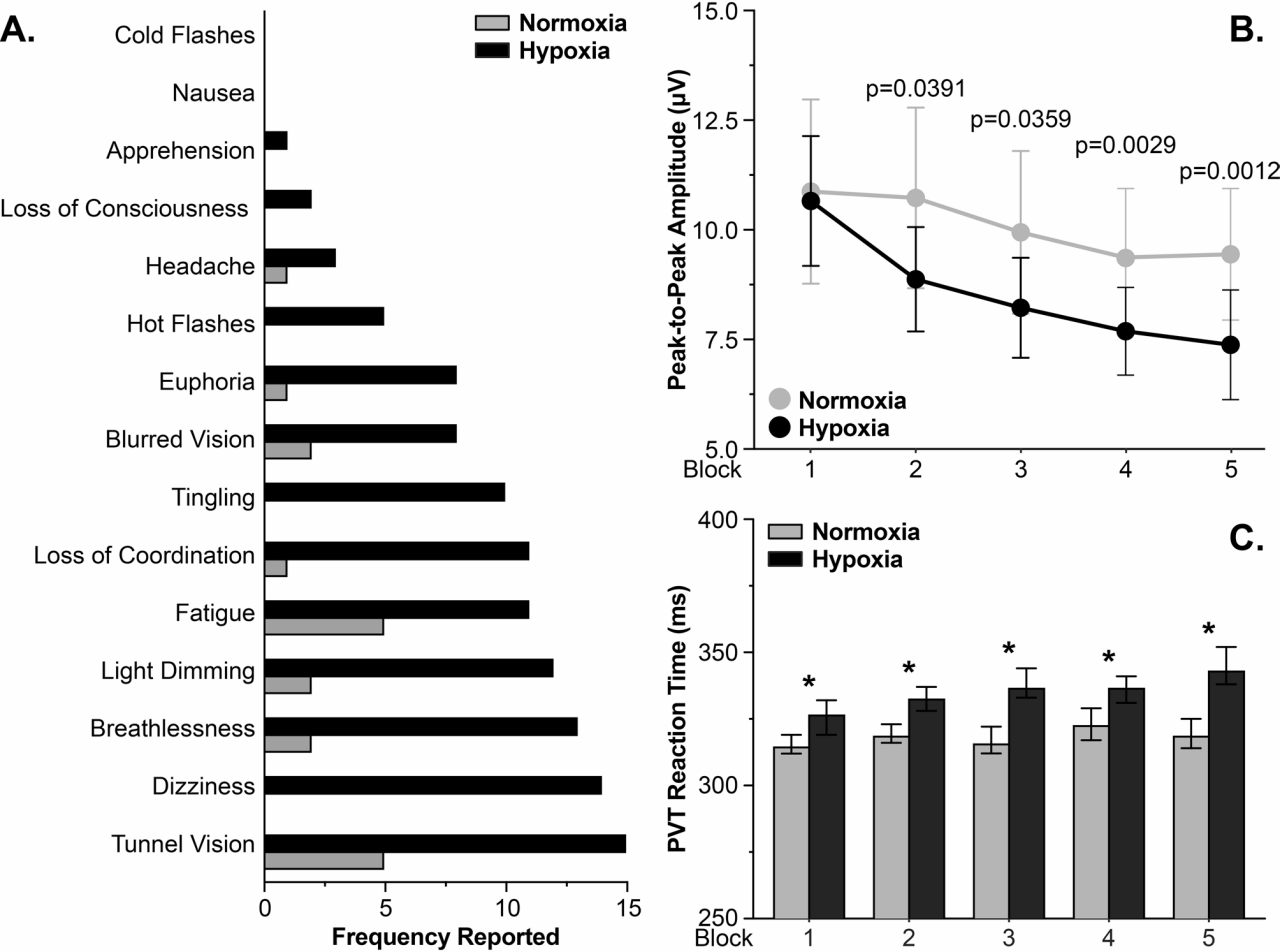
Reported and measured response to normobaric hypoxia. **(A)** A summary of the frequency of reported perceived symptomology based on the hypoxia symptom questionnaire following each exposure, normoxia and hypoxia. **(B)** A plot of the peak-to-peak (P2P) amplitude from EEG data, by block, acquired throughout normoxic and hypoxic conditions. **(C)** A plot of the Psychomotor Vigilance Task (PVT) median reaction times, by block, for normoxia and hypoxia exposures. * indicate *p* < 0.0001 and all error bars signify the 95% confidence interval. The data illustrate normobaric hypoxia induced significant effects on participant’s perceived physiology, brain activity, and cognition

Finally, the hypoxia symptom questionnaire (HSQ) was provided to participants following each exposure to assess an individual’s perceived symptoms. Figure 2A indicates hypoxia induced a higher frequency of reported symptoms (black bars) for nearly all attributes evaluated. Furthermore, 100% of participants (22/22) reported experiencing at least one symptom during the hypoxic exposures, while 45% (10/22) did not report experiencing a single symptom during the normoxia exposures. The most frequently reported symptoms during the hypoxia exposures were tunnel vision, dizziness, and breathlessness, which are consistent with prior studies on hypoxia symptoms [8, 36]. Overall, the results suggest a whole-body physiological response, as a decrease in minSpO_2_, variable HR, changes in blood chemistry, and differences in reported symptomatology were induced among individuals exposed to normobaric hypoxia conditions.

### Brain activity & cognitive function

ERPs recorded during an “oddball” paradigm were evaluated for the difference between standard and deviant stimuli (i.e., MMN, P3a, and peak-to-peak amplitude). Please see Supplemental Data 6 A for a depiction of the ERP amplitudes measured. The data show a non-significant difference between the overall P3a (*p* = 0.1062) and MMN (*p* = 0.6132) amplitudes for normoxia compared to hypoxia (Supplemental Data 6B & 6D). When the values are parsed by blocks and exposure, the P3a amplitudes at Block 5 show a significant reduction (*p* = 0.0176) while the MMN amplitudes show no significant difference between conditions (*p* ≥ 0.1129, Supplemental Data 6 C & 6E). Finally, when evaluating the change in waveform amplitudes (peak-to-peak, P2P, the difference between MMN and P3a, Supplemental Data 6 A), a significant change in overall P2P amplitude is observed (*p* = 0.0199) when comparing hypoxic to normoxic exposures (Supplemental Data 6 F). Additionally, when resolved by block, there is a significant reduction in the P2P amplitudes compared to the normoxic exposures in the hypoxia condition at Blocks 2, 3, 4, and 5 (*p* ≤ 0.0391, Fig. 2B). These data indicate hypoxia exposures induce a significant change in auditory information processing when compared to normoxic exposures.

The results from Fig. 2B indicate a significant disruption to the ERPs measured here in response to hypoxia. To further evaluate the impact of hypoxia on cognitive function, three cognitive performance tests were evaluated: the Psychomotor Vigilance Task (PVT), the Change Signal Task (CST), and the Digit Symbol Substitution Task (DSST). The data indicate that the PVT test showed the greatest difference when evaluating between the hypoxia and normoxia data (Fig. 2C, Supplemental Data 7). For instance, the overall PVT response time and the PVT response times parsed by testing block showed significant increases in the hypoxia group (*p* < 0.0001, Fig. 2C **&** Supplemental Data 7 A). The results suggest a reduction in participant’s ability to sustain attention and respond quickly to visual stimuli. While the PVT data for lapse rate, miss rate, and false starts are not described here, the data in Fig. 2C **&** Supplemental Data 7 A indicate a possible cognitive decline associated with the hypoxic condition when compared to normoxia exposures.

Although PVT showed the largest effect when comparing hypoxia to normoxia, the CST and DSST tests were also evaluated. First, the CST response time illustrates an overall significant reduction in reaction time (*p* = 0.0468) during the hypoxic exposure with Block 2 showing a significant difference (*p* = 0.0351), hypoxia to normoxia (Supplemental Data 7B & 7 C). Additionally, the overall error rate (responding with the incorrect direction in non-change signal trials and change signal trials) demonstrates a significant increase in errors in the hypoxia group (*p* = 0.0002) while no specific blocks show a significant difference (Supplemental Data 7D & 7E). The DSST data illustrates no significant difference in overall or by block reaction times (Supplemental Data 8 A & 8B) while the overall error rate (*p* = 0.0149) and Block 5 (*p* = 0.0319) show significant differences between hypoxia and normoxia (Supplemental Data 8 C & 8D). It is plausible that due to the large variability in the measurements the CST and DSST would show greater significance with an increased sample size (Supplemental Data 7 & 8). Collectively, the results illustrate a change in brain electrical pulses and a reduction in cognition associated with hypoxic exposures, specifically increased incorrect responses in the CST and decreased response times in the PVT.

To determine if the measured minSpO_2_ of an individual correlated with the neuronal effects observed, i.e. changes in EEG P2P and PVT reaction times, Pearson correlation coefficients were calculated across exposure blocks and conditions. Supplemental Data 9 shows low correlation among any of the attributes in either the normoxia (Top, -0.18 ≤ ρ ≤ 0.28) or hypoxia (Bottom, -0.54 ≤ ρ ≤ 0.21) exposure conditions. While the mean P2P amplitude approaches a significant negative correlation (-0.70 ≤ ρ ≤ 0.70) with minSpO_2_ at block 4 (ρ=-0.54), the data suggest that the minSpO_2_ does not correlate directly with brain activity and cognitive function (Supplemental Data 9).

### Exhaled Nitric Oxide & PTR-MS Data

Due to the relatively large quantities of literature surrounding fractional exhaled nitric oxide (FeNO) and hypoxemia, FeNO was evaluated within each testing block in addition to before and after the exposures [37–42]. Supplemental Data 10 A & 10B show the overall and time-resolved FeNO measurements for both hypoxia and normoxia exposures. The data indicate no significant difference in FeNo was observed (*p* > 0.05). These data support the hypothesis that there is no significant change in exhaled nitric oxide under normobaric hypoxia conditions [40–42].

**Fig. 3:**
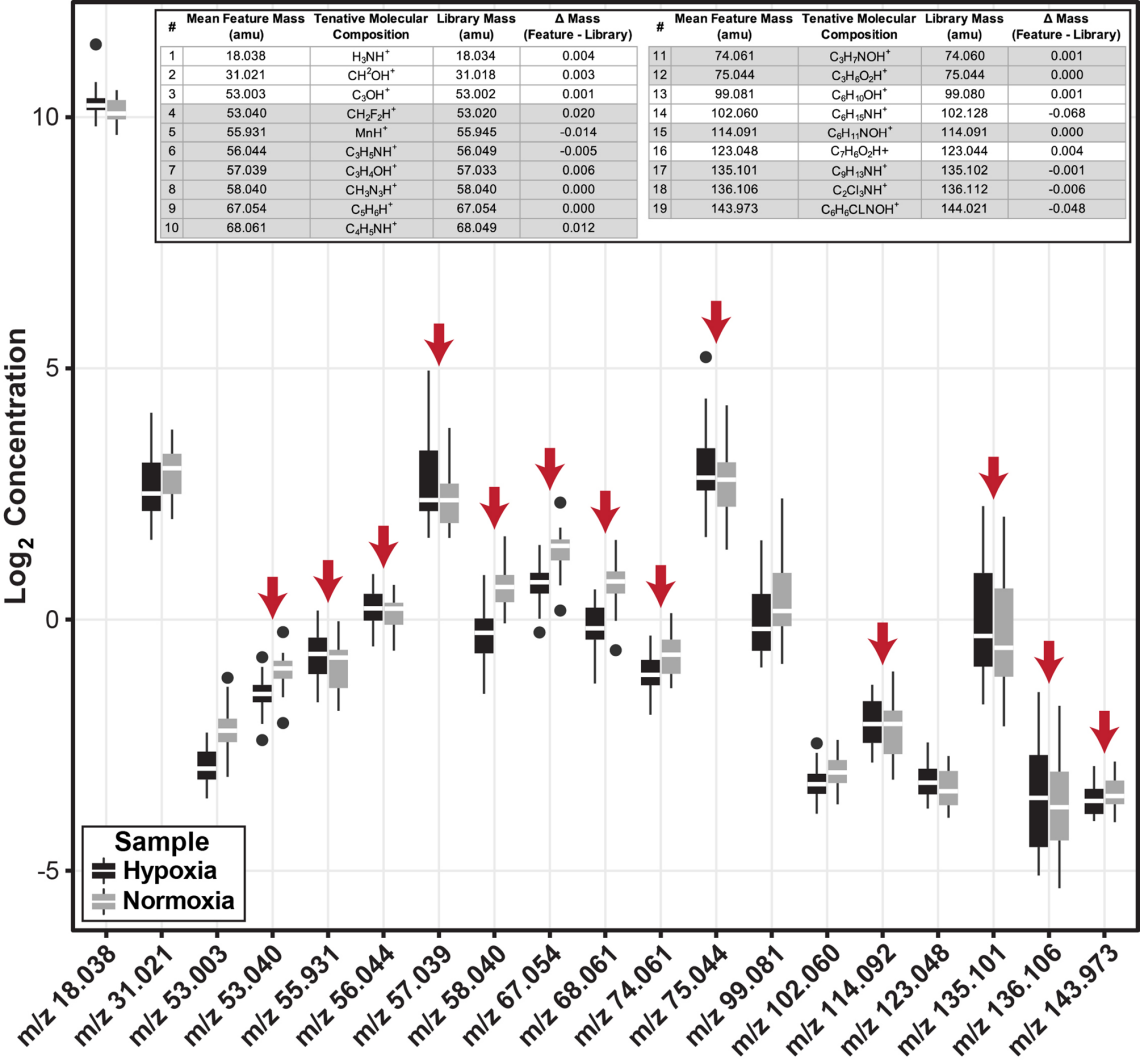
Changes in exhaled breath feature concentrations in response to normobaric hypoxia exposures. A plot of the normalized log_2_ concentrations (ppb) of 19 exhaled breath features found to be statistically different between normoxic and hypoxic exposures at Block 3. The data show 13 features are enriched in the breath and differ in concentration for hypoxia compared to normoxia. Red arrows indicate the 13 exhaled breath features used in further analyses that are statistically increased in exhaled breath when compared to background at Block 3 (Supplemental Data 12). Inset: tentative molecular composition of the 19 exhaled breath features based on accurate mass. Highlighted features are those related to the reduced model within Fig. 4

**Fig. 4:**
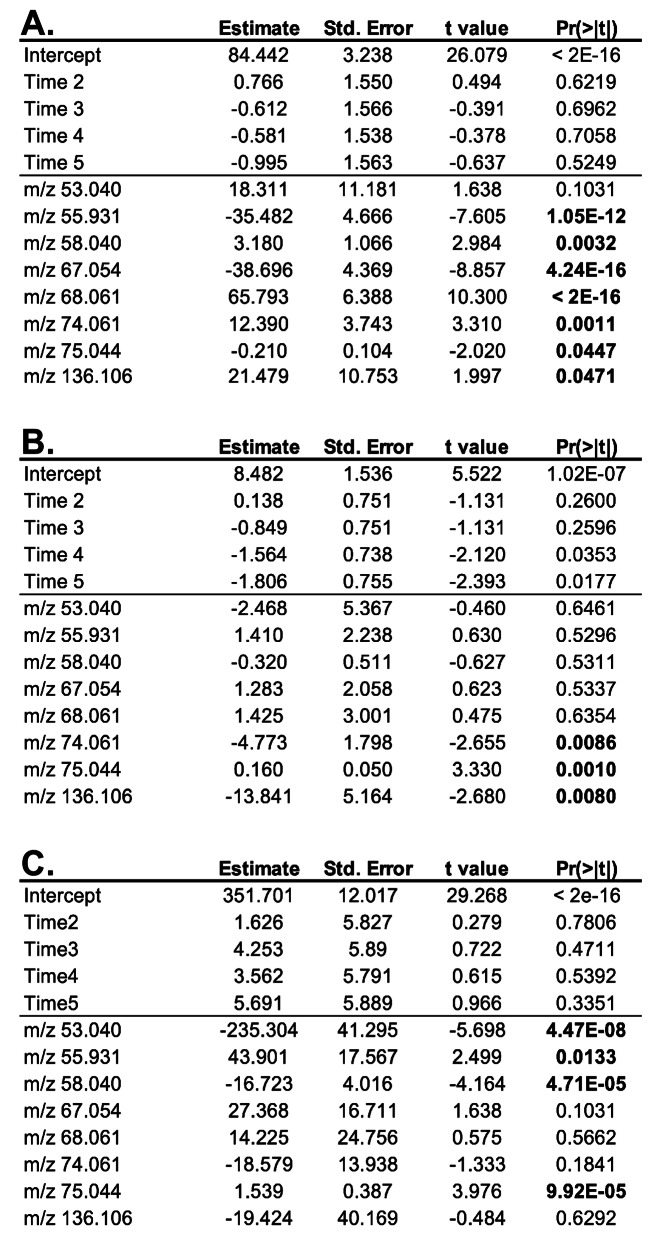
Concentrations of 8 exhaled breath features indicate changes in the individual’s response to acute hypoxia exposures. Through linear mixed modeling, **(A)** SpO_2_, **(B)** EEG P2P amplitude, and **(C)** PVT reaction time changes from normobaric hypoxia exposures. Bold p-values indicate significance (*p* < 0.05). The data indicate the possibility for PTR-MS exhaled breath feature concentrations to indicate changes among individuals in response to normobaric hypoxia

In addition to FeNO measurements, exhaled breath was directly measured with two breaths acquired at the beginning of each testing block, approximately 30s apart (Fig. 1D). See Supplemental Data 11 A for a representative PTR-MS time course acquisition, illustrated by plotting isoprene (m/z 69), and a representative spectra from a single exhalation (Supplemental Data 11B). To evaluate the PTR-MS data for exhaled features that change in response to hypoxia, 135 feature concentrations were manually extracted from more than 110 individual data files and averaged by block. Please refer to Supplemental Data 4 for an Excel file of the determined concentrations and Supplemental Data 11 C for a summary of the data workflow. As minSpO_2_ is the primary measurement for illustrating a hypoxemic effect, Block 3, the block where hypoxia no longer induced a significant change in SpO_2_, was utilized to evaluate and down select features. At Block 3, there were 19 features with a statistically significant log_2_ concentration (ppb) difference between hypoxia and normoxia (Fig. 3). To further evaluate the results with respect to background, the 19 down selected feature log_2_ concentrations (ppb) from breath were plotted against the background values (Supplemental Data 12 A & 12B). Statistical evaluation was conducted to identify features among the 19 down selected features showing a significant increase (*p* < 0.05) in concentration in breath compared to background within both exposure conditions. Identified by red arrows in Figs. 3 and 13 features were enriched in breath while illustrating a significant difference between hypoxic and normoxic exposure breath at Block 3. Using the measured mass associated with each of the 13 significant features, a tentative molecular composition was determined (Fig. 3**Inset**). The inset features highlighted in grey are those found in the reduced model within Fig. 4. While the analysis did not account for all time points measured, the results yielded a reduced list of features that are significantly changed during hypoxic exposures for further predictive modeling. Collectively, exhaled breath volatile analysis shows several features are differentially expressed in the hypoxic condition, providing plausibility for the use of exhaled volatiles for determination of hypoxemic onset.

### Exhaled breath for prediction of neuronal functional decline

As brain activity and cognitive function are extremely difficult to estimate directly in the field and minSpO_2_ did not directly correlate with cognitive decrement (Supplemental Data 9), an alternative means for predicting hypoxia-induced decline is advantageous. To determine if exhaled breath feature concentrations could be indicative of these data types, the 13 significant features identified as enriched in breath and differentially expressed in hypoxia (Fig. 3) were evaluated by linear mixed modeling (LMM). LMM was utilized to account for random effects associated with the large participant variability and interpersonal correlation of measures across time points. The 13-feature input was further reduced using a bidirectional stepwise function to determine the best fit of the features that relate to minSpO_2_. Figure 4A provides a table of the results from the reduced model. The data illustrate 8 feature concentrations are significantly indicative of the minSpO_2_ across all time points. Although minSpO_2_ is interesting, the primary goal is to evaluate the use of exhaled breath on brain activity and cognitive performance. The 13 features within the reduced model (Fig. 4A) were evaluated to determine the relationship to change in the P2P EEG amplitude. Figure 4B shows 3 features were significantly indicative of the changes in P2P amplitudes observed. Similarly, when the reduced model was applied to the PTV reaction times among blocks, 3 features’ concentrations are significantly indicative of the increased PVT reaction times found during the hypoxia exposures. These data illustrate the potential for exhaled breath volatiles to relate to physio/cognitive measurements through linear mixed modeling.

## Discussion

As hypoxia-like events remain a threat to USAF and Navy USN pilots’ safety, early detection of hypoxia-related impairment remains paramount to protecting pilot lives. As illustrated in Supplemental Data 9, the measurement of an individual’s SpO_2_ alone does not significantly correlate with changes in brain activity and cognitive function resulting from hypoxia exposure, as assessed here. These data suggest other means, beyond SpO_2_ monitoring, are needed to fulfill this requirement. It is hypothesized that changes in exhaled breath VOC content could meet this need. Preliminary studies supporting this hypothesis have been conducted [11, 12]. Data collected by Harshman et al., utilizing mask-on exposures, show abundance of several volatiles, such as Isoprene, Pentanal, and 2-Pentanone, was modified with respect to hypoxic insult [11, 12]. While mask-based exposures are more operationally relevant to USAF and USN pilots, mask-based breathing is often labored and difficult independent of the O_2_ content provided to the individual, plausibly influencing the results [43–45]. The study described here was designed to determine changes in the exhaled VOCs resulting from hypoxic exposure independent of a flight mask.

The data in Fig. 3 show exhaled breath features with concentrations that significantly change in response to mask-free normobaric hypoxia exposures in a controlled environment. While noteworthy, using exhaled breath VOCs to estimate changes in SpO_2_ is not the most operationally relevant issue to the USAF and USN. Of most concern to aircrew and commanders are changes in cognition, hypothesized to result from changes in SpO_2_, that could ultimately lead to in-flight safety issues. As individuals respond to hypoxia exposures differently, i.e. some individuals function at a high cognitive performance with low SpO_2_ and vice versa, the data shown in Fig. 4 illustrate several features that are indicative of changes in brain activity (Fig. 4B) and cognitive performance (Fig. 4C). These results represent the first of its kind by linking changes in exhaled breath volatile compound concentrations to hypoxia-induced changes in the brain. While future work will be required to validate the results, these data establish a novel set of hypoxia-related volatiles from exhaled breath for further development for flight crew performance monitoring with the goal of developing small, real-time sensing elements that will fit within the exhalation port of a flight mask.

The results in Fig. 3 show 19 features that are statistically different between the hypoxic and normoxic conditions, with 13 of those features having higher concentrations in the exhaled breath compared to background at Block 3. Of the 13 features from Fig. 3, none of the m/z values overlap with the theoretical m/z values of compounds previously identified to change in response to hypoxemia by Harshman et al. (Isoprene m/z 69.07, Pentanal m/z 87.08 & 69.07, 4-Butyrolactone m/z 87.04, 2-Pentanone m/z 87.08, 2-Hexanone m/z 101.10, 2-Cyclopenten-1-one m/z 83.05, and 2-Heptanone m/z 115.11) [11]. The lack of consistency among these previous studies and the current study is likely due to several reasons [46]. First, as discussed previously, the data presented here were collected within the ROBE, removing the potentially confounding factor of the flight mask causing changes in response to hypoxia derived from the exposure methodology. For instance, isoprene measured within this study did not illustrate a significant difference between hypoxia and normoxia, while evidence in the literature suggests a gradual decrease of exhaled isoprene from individuals wearing a medical facemask suggested to result from hypoxemia induced peripheral vasoconstriction [47]. The confounding results between this and previous studies highlight the dynamics of pulmonary ventilation/perfusion and compartmental release of VOCs under hypoxic conditions in the presence or absence of a mask, where partial re-breathing due to a mask or physiological response without a mask could play a significant role in exhaled data [48]. Second, the study length and severity of exposures were modified within the current manuscript compared to historical studies. For example, previous studies were conducted at O_2_ equivalent levels of 25,000ft for 5 min while the current study was conducted at 17,500ft O_2_ equivalent for 45 min [11, 12]. The lower altitude equivalent oxygen was purposefully selected to induce a hypoxic response but allow for a prolonged sampling time to gain a better understanding of the body’s reaction to low oxygen. Next, previous studies utilized thermal desorption gas chromatography mass spectrometry (TD-GC-MS) for analysis while collecting volatiles serially throughout the short exposure time [11, 12]. While advantageous due to preconcentration of volatiles prior to analysis, a level of specificity is added by using adsorbent materials, i.e. detection relies on binding of the compound to the adsorbent. The PTR-MS is a real-time measurement device, with specificity of its own relating to proton transfer of hydronium (H_3_O^+^); therefore, it is plausible that results of the two methodologies do not overlap due to specificity. Finally, 2-Pentanone and Pentanal have isobaric intact masses within the PTR-MS spectra. For example, the mass of 2-Pentanone and Pentanal are isobars at m/z 87.08. It is hypothesized that variable concentrations of these compounds could plausibly mask significant changes due to the overlapping masses.

In addition to issues with isobars, as described above, the PTR-MS does not yield tentative compound identifications, as afforded by traditional electron impact GC-MS, beyond chemical composition related to the accurate mass (level 3 identifications) [49]. For example, a single feature, 75.044 m/z, was found to be related to the three-performance metrics investigated (SpO_2_, PVT, P2P) (Fig. 4). When 75.044 m/z was searched in the Ionicon Ioni Database within the PTR-MS Viewer Software, three possible chemical names were provided based on a C_3_H_6_O_2_ (H^+^) composition with a theoretical mass of 75.04406 m/z. While all three tentative chemicals are short chain fatty acids (acetic acid, methyl ester, formic acid, ethyl ester, and propanoic acid), identifying which specific compound, or combination of these compounds, are dysregulated during hypoxia is impossible from these data. As a result, the features in the reduced model’s dataset will require orthogonal detection methodologies, such as GC-MS or GCxGC-MS, to accurately identify the VOCs of interest.

Finally, there is contradictory data indicating there may or may not be changes in exhaled nitric oxide in hypoxic individuals. For example, Brown et al., Busch et al., and Duplain et al., independently showed significant decreases in exhaled NO in individuals at high altitude, i.e. hypobaric hypoxia [37–39]. These data would follow the plausible mechanism suggesting decreased exhaled NO is a result of a lack of available O_2_ required for production [50]. However, Hemminsson and Linnausson and Donnelly et al. have shown no change in exhaled NO among individuals experiencing normobaric hypoxia [40, 41]. Furthermore, MacInnis et al. illustrated that at 1 h of a 4550 m (~ 15,000ft) normobaric hypoxia exposure, there was no change in exhaled NO [42]. The data in Supplemental Data 10 support the O_2_ independent model of exhaled NO under normobaric hypoxic conditions. It is of note that the FeNO measured was “multiple breaths mode” where concentration was not determined at 50 mL s^-1^, as outlined by the American Thoracic Society, which may have impacted the results [51].

## Conclusion

The results indicate further support for the use of exhaled breath volatile compound profiling for non-invasive human performance monitoring. The results show the first of its kind data supporting differences in exhaled breath volatile concentrations, indicating changes in brain activity and cognition derived from hypoxic insult. However, additional studies will be required to validate results with novel sampling and analytical approaches, within more operationally relevant simulated environments, to fully identify features of interest from the PTR results.

## Electronic supplementary material

Below is the link to the electronic supplementary material.

Supplementary Material 1

Supplementary Material 2

## Data Availability

The datasets generated and/or analyzed during the current study are available, as per the current guidance, at the following link https://storage.googleapis.com/afrl-il2-backup-gcs-padata-xtb5/2025.01.13/afrl.2024.6964/hypoxia-data-AFRL-2024-6964.zip

## Declarations

### Disclaimer

Imagery in this document are property of the U.S. Air Force. The views expressed are those of the authors and do not reflect the official guidance or position of the United States Government, the Department of Defense, the United States Air Force, or the United States Space Force.
