# Supplementary material for: Estimating hypoxia-induced brain dysfunction and cognitive decline through exhaled breath monitoring
Source: Respir Res. 2025 Jun 13;26:215. doi: 10.1186/s12931-025-03296-5 (PMC12164151; doi:10.1186/s12931-025-03296-5)

Supplemental Data

**SUPPLEMENTAL DATA 1:** **A)** A representative plot of the Reduced Oxygen Breathing Environment’s (ROBE) measured O_2_ throughout an exposure. **B)** A box plot of the measured O_2_ from all exposures. All measurements were performed at 1Hz­ with a real-time sensor within a MultiRae Pro pump (Honeywell, San Jose, CA, USA) operated at approximately 270 mL min^-1^.

**
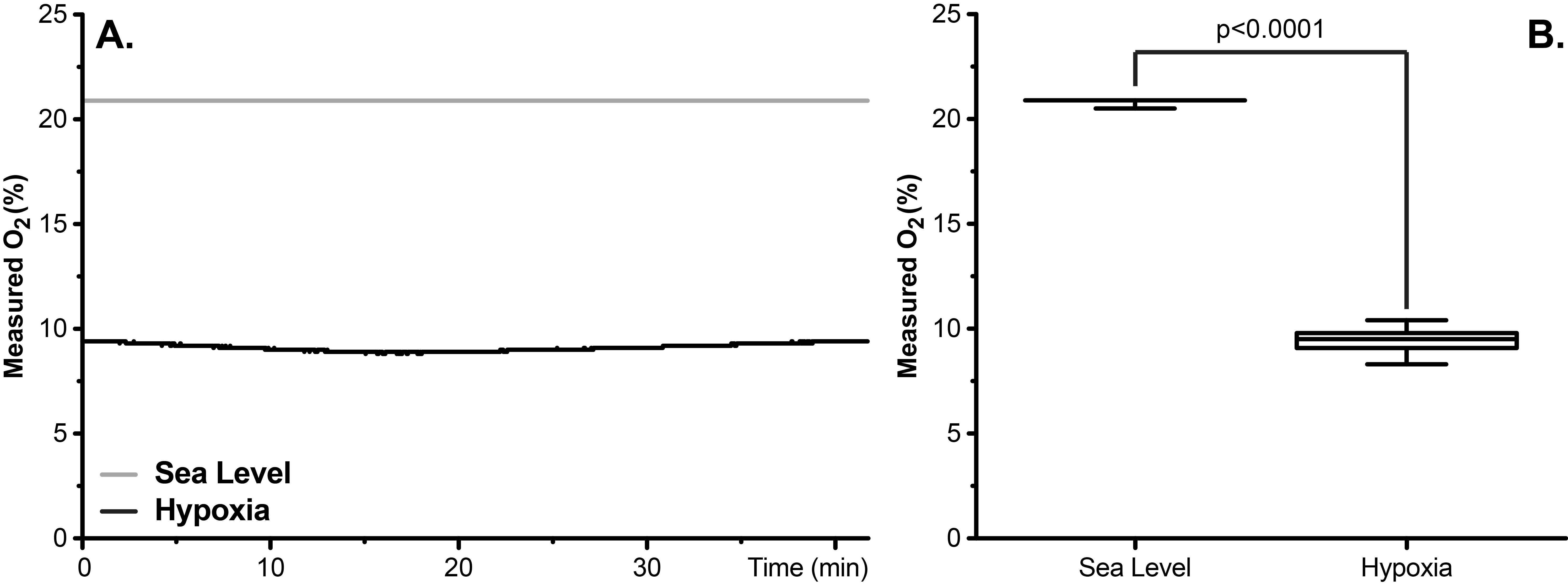
**

**SUPPLEMENTAL DATA 2:** **A)** A box plot of the minimum oxygen saturation (minSpO_2_) from sea level and hypoxia exposures. **B)** A representative trace of the measured heart rate of an individual exposed to sea level and hypoxia. **C)** A box plot of the maximum heart rate from sea level and hypoxia exposures. Box plot error bars indicate the min and max values. **D)** A bar chart of the maximum heart rate by block from sea level and hypoxia exposures where * indicates p<0.05 comparing sea level to hypoxia and error bars represent the 95% confidence interval.

**
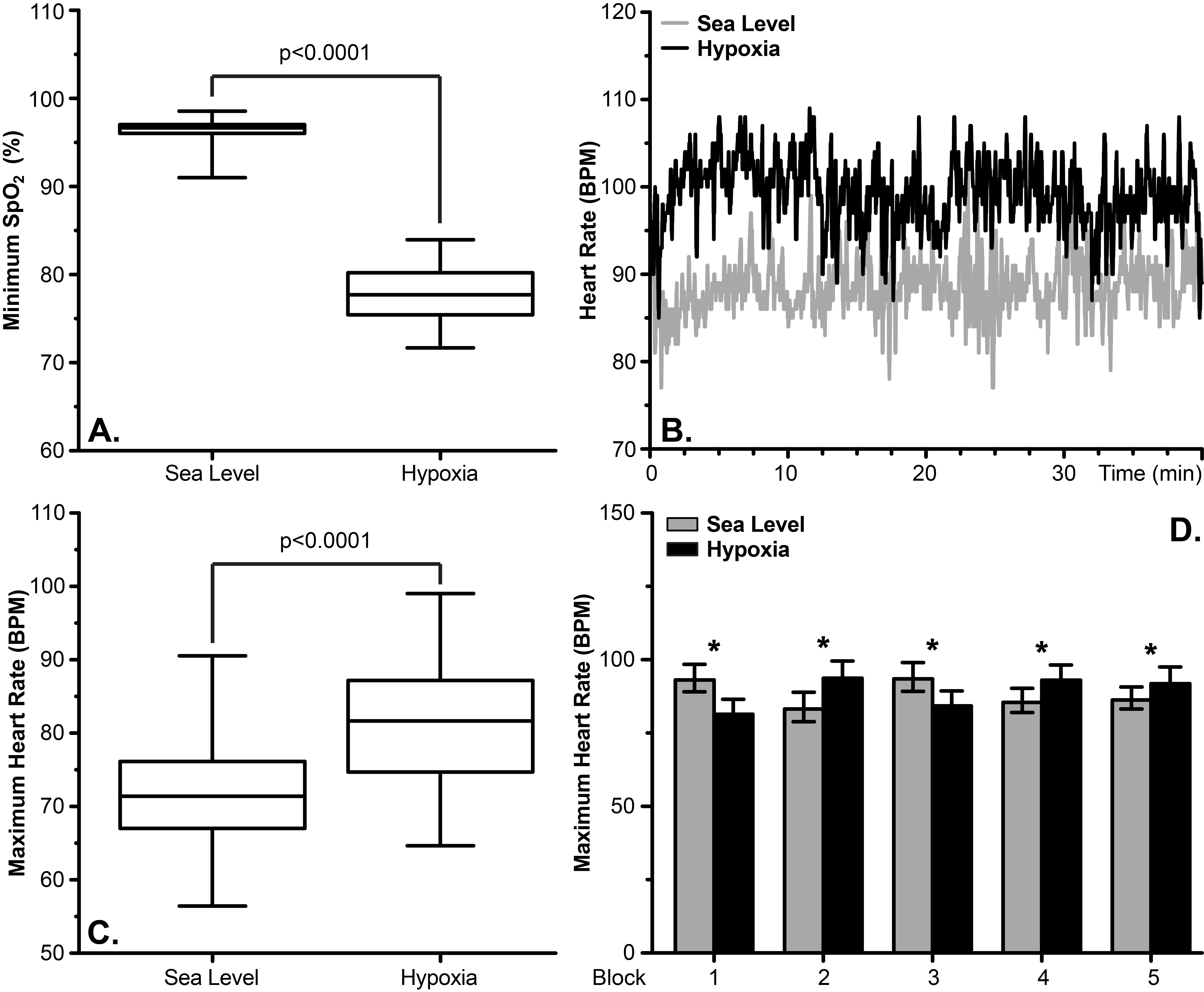
**

**SUPPLEMENTAL DATA 3:** A representative example of the hydronium (21.02 m/z) over the entire exposure.

**
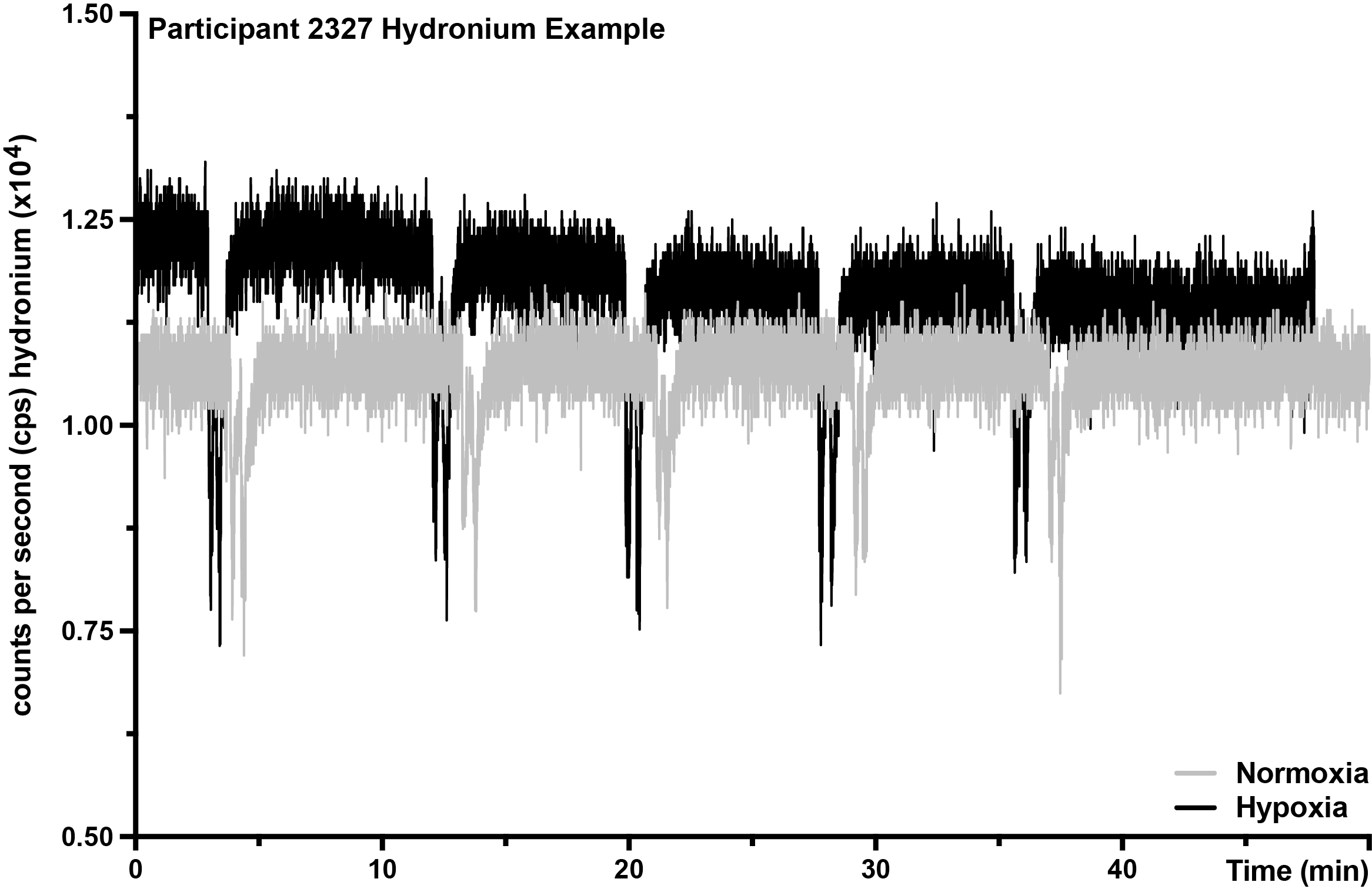
**

**SUPPLEMENTAL DATA 4:** An .xlsx file containing the determined feature concentrations (ppb) for each sample and the LOD values for each feature.

**SUPPLEMENTAL DATA 5:** Post-Pre delta values for blood **A)** gases (partial pressure of carbon dioxide (PCO_2_), partial pressure of oxygen (PO_2_), total carbon dioxide (TCO_2_), bicarbonate (HCO_3_), base excess (BE), oxygen saturation (sO_2_)) and pH and **B)** chemistry from the iSTAT point of care device. * indicates p<0.05 comparing sea level to hypoxia deltas and error bars represent the 95% confidence interval.


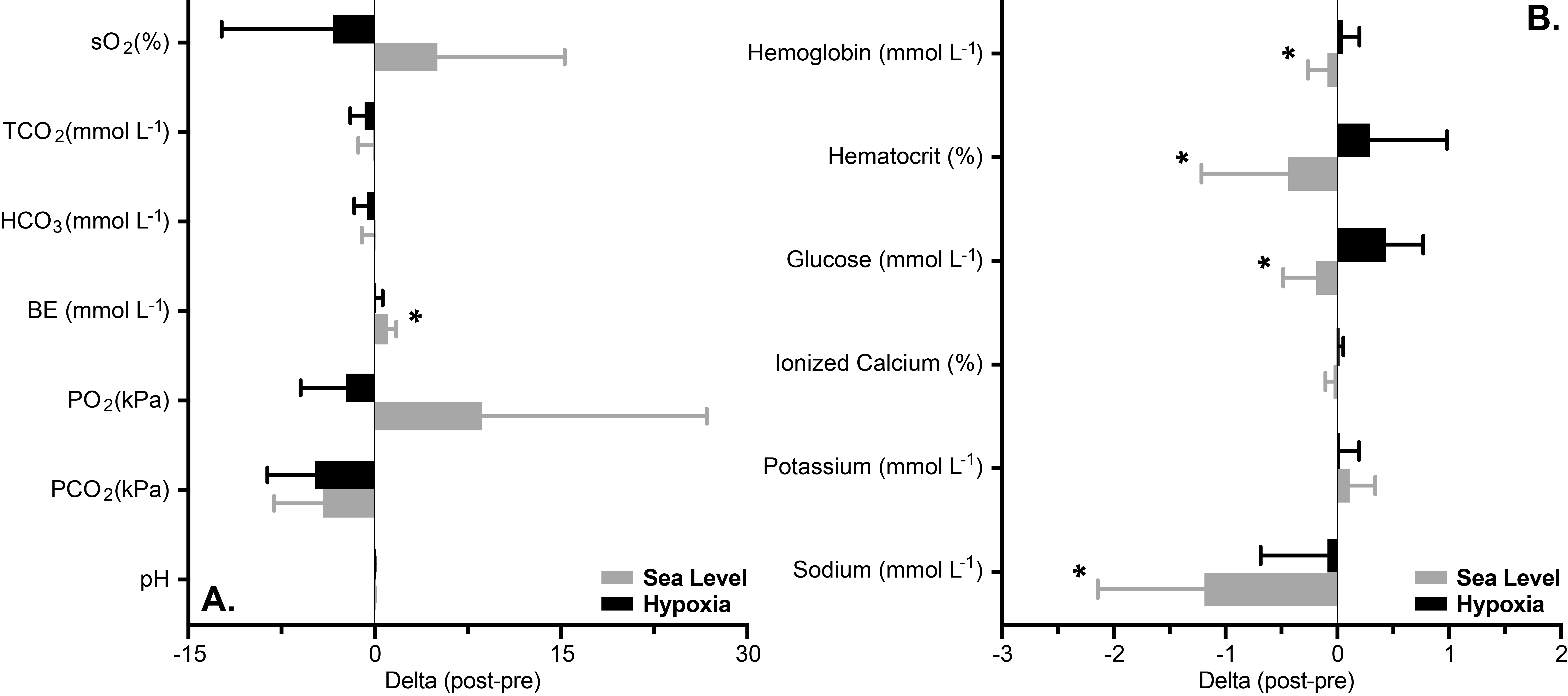


**SUPPLEMENTAL DATA 6: A)** A representative electroencephalogram depicting the measured values of the EEG data (MMN, mismatch negativity). **B)** A bar graph showing the overall P3a amplitudes from sea level and hypoxia exposures. **C)** A graph showing the P3a amplitudes by testing block from sea level and hypoxia exposures. **D)** A bar graph showing the overall MMN amplitudes from sea level and hypoxia exposures. **E)** A graph showing the MMN amplitudes by testing block from sea level and hypoxia exposures. **F)** A bar graph showing the overall peak-to-peak amplitude values from sea level and hypoxia exposures. All error bars indicate the 95% confidence interval.


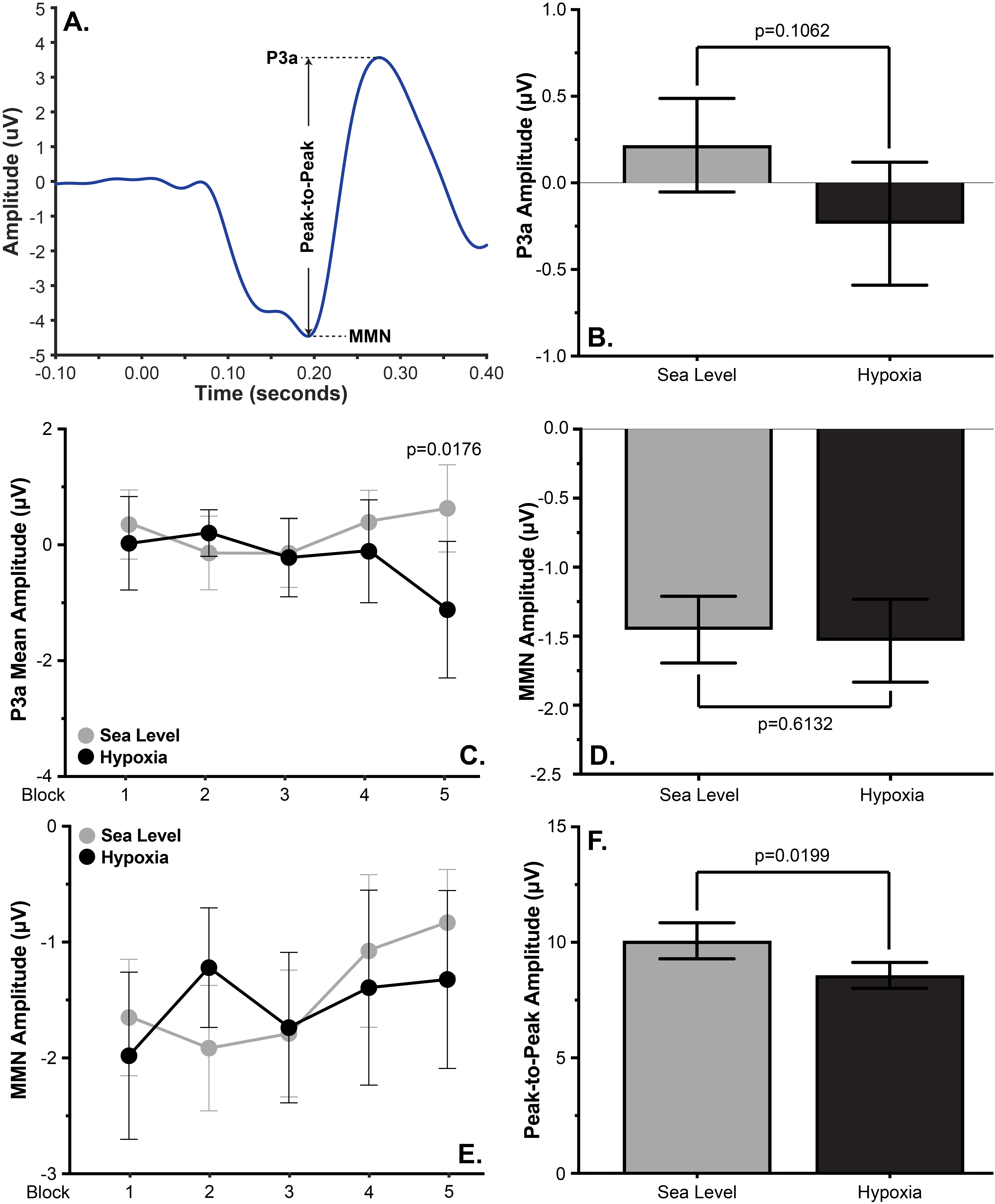


**SUPPLEMENTAL DATA 7: A)** A graph showing the overall Psychomotor Vigilance Task (PVT) reaction time values from sea level and hypoxia exposures. **B)** A graph showing the overall Change Signal Task (CST) reaction times from sea level and hypoxia exposures. **C)** A graph showing the CST reaction time values by testing block from sea level and hypoxia exposures. **D)** A graph showing the overall CST error rate (%) values from sea level and hypoxia exposures. **E)** A graph showing the CST error rate (%) values by testing block from sea level and hypoxia exposures (all p>0.05). All error bars indicate the 95% confidence interval.


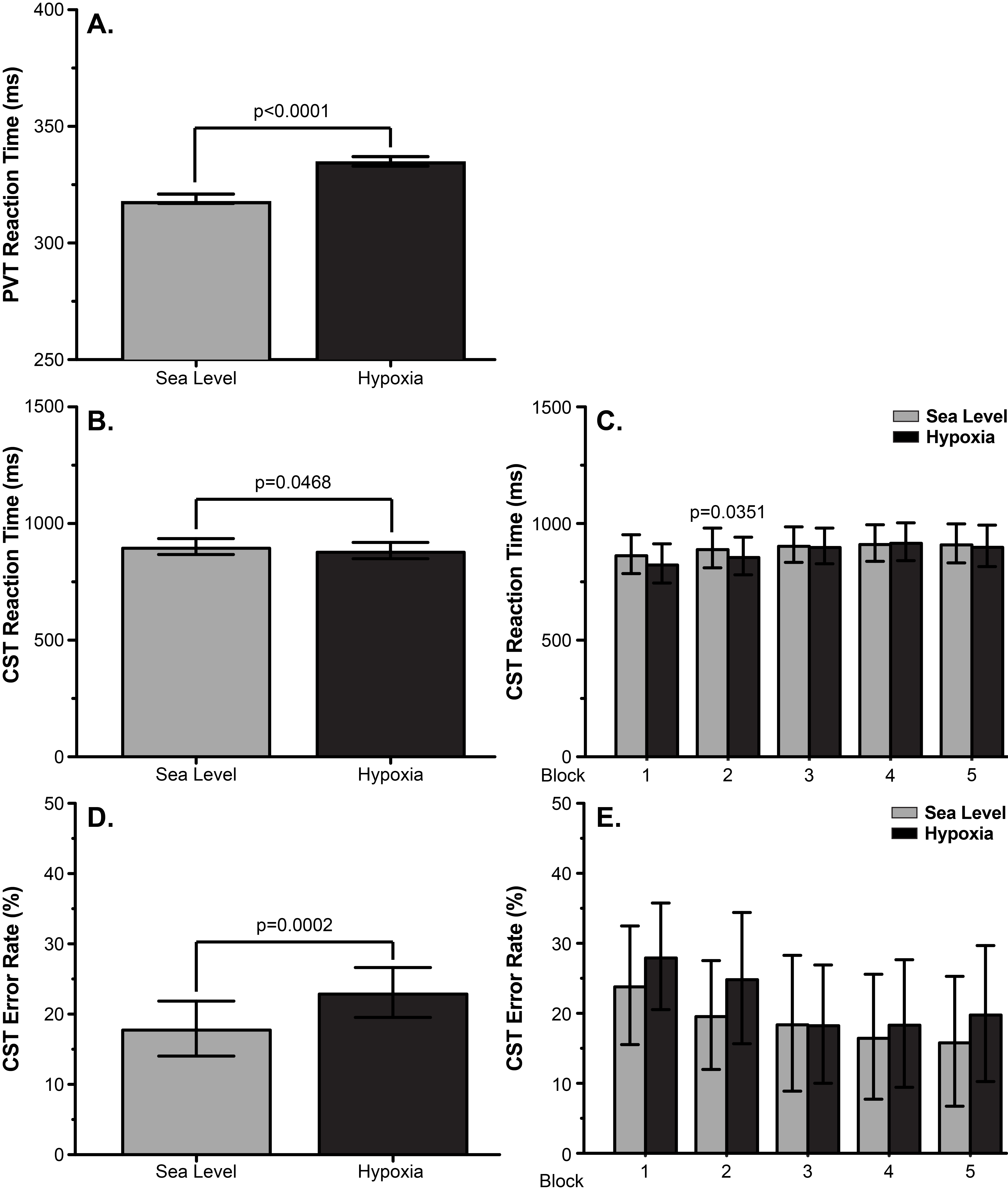


**SUPPLEMENTAL DATA 8: A)** A graph showing the overall Digit Symbol Substitution Task (DSST) reaction time values from sea level and hypoxia exposures. **B)** A graph showing the DSST reaction time values by testing block from sea level and hypoxia exposures (all p>0.05). **C)** A graph showing the overall DSST error rate (%) values from sea level and hypoxia exposures. **D)** A graph showing the DSST error rate (%) values by testing block from sea level and hypoxia exposures. All error bars indicate the 95% confidence interval.

**
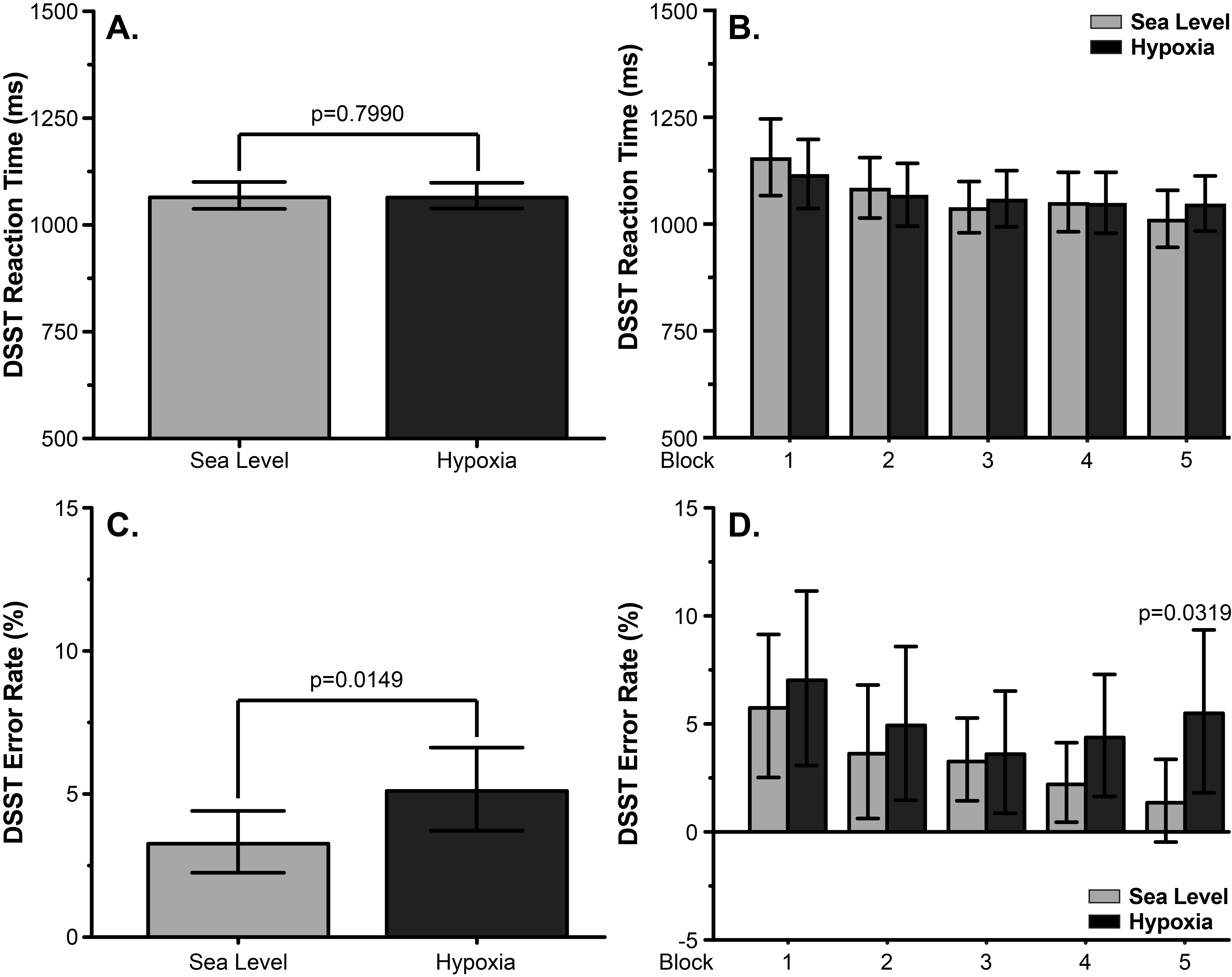
**

**SUPPLEMENTAL DATA 9:** Pearson correlation of oxygen saturation (SpO_2_), EEG peak-to-peak amplitude (P2P), and psychomotor vigilance reaction time (PVT RT) by block and oxygen exposure. Correlation values >0.70 and <0.70 were considered significant.

**
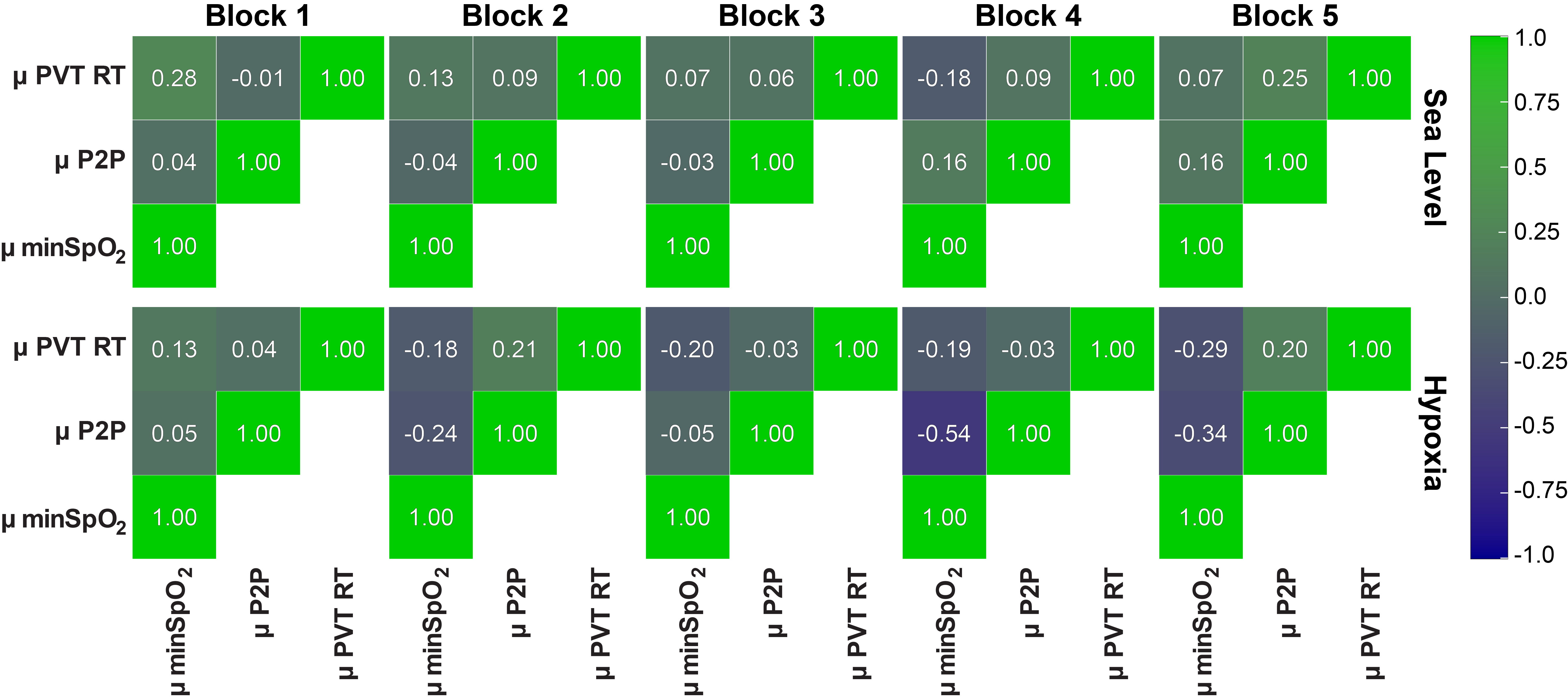
**

**SUPPLEMENTAL DATA 10:** **A)** A bar graph of the overall fractional exhaled nitric oxide (FeNO) values from the exposures. **B)** A plot of the exhaled fractional exhaled nitric oxide (FeNO) from before, during, and after sea level and hypoxia exposures (all p>0.05). Error bars represent the 95% confidence interval.

**
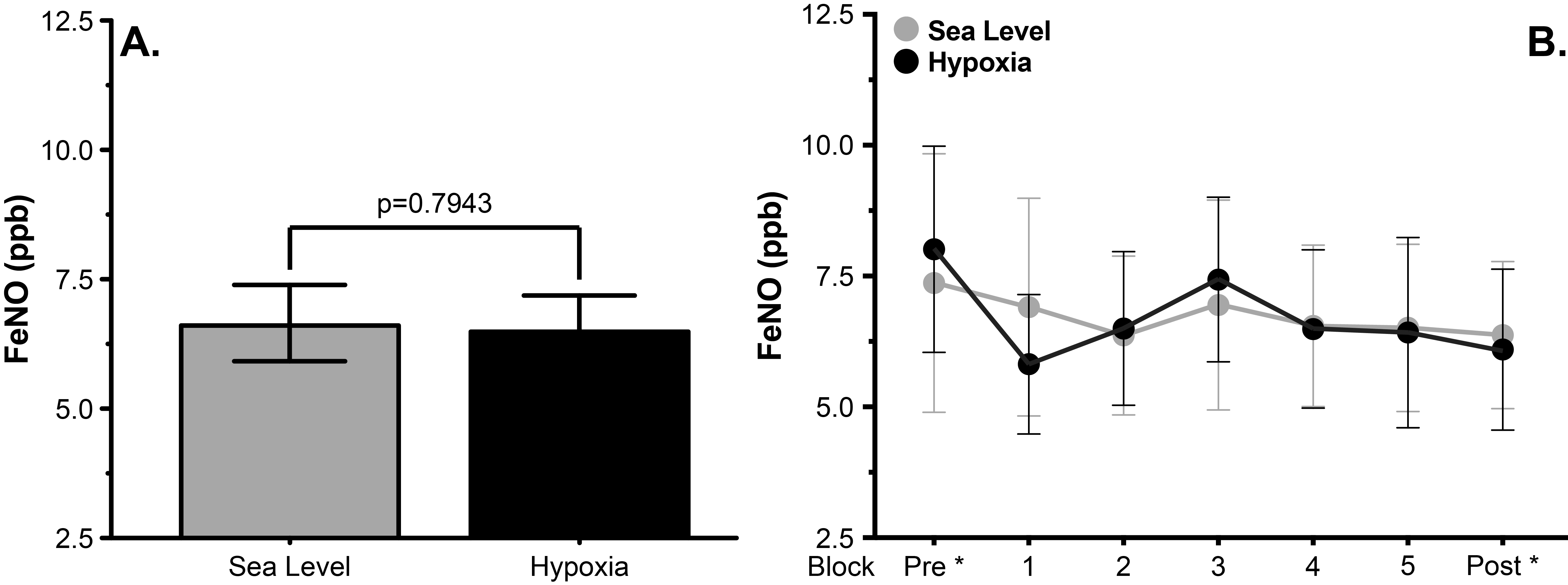
**

**SUPPLEMENTAL DATA 11:** **A)** A representative PTR-MS time course of isoprene (m/z 69), illustrating the breath sampling by block over the entire exposure. **B)** A representative PTR-MS spectra taken from an exhaled breath sample during an exposure with an inset zoomed-in spectra (red box). **C)** The workflow utilized for processing of the Proton Transfer Reaction Mass Spectrometry (PTR-MS) data.


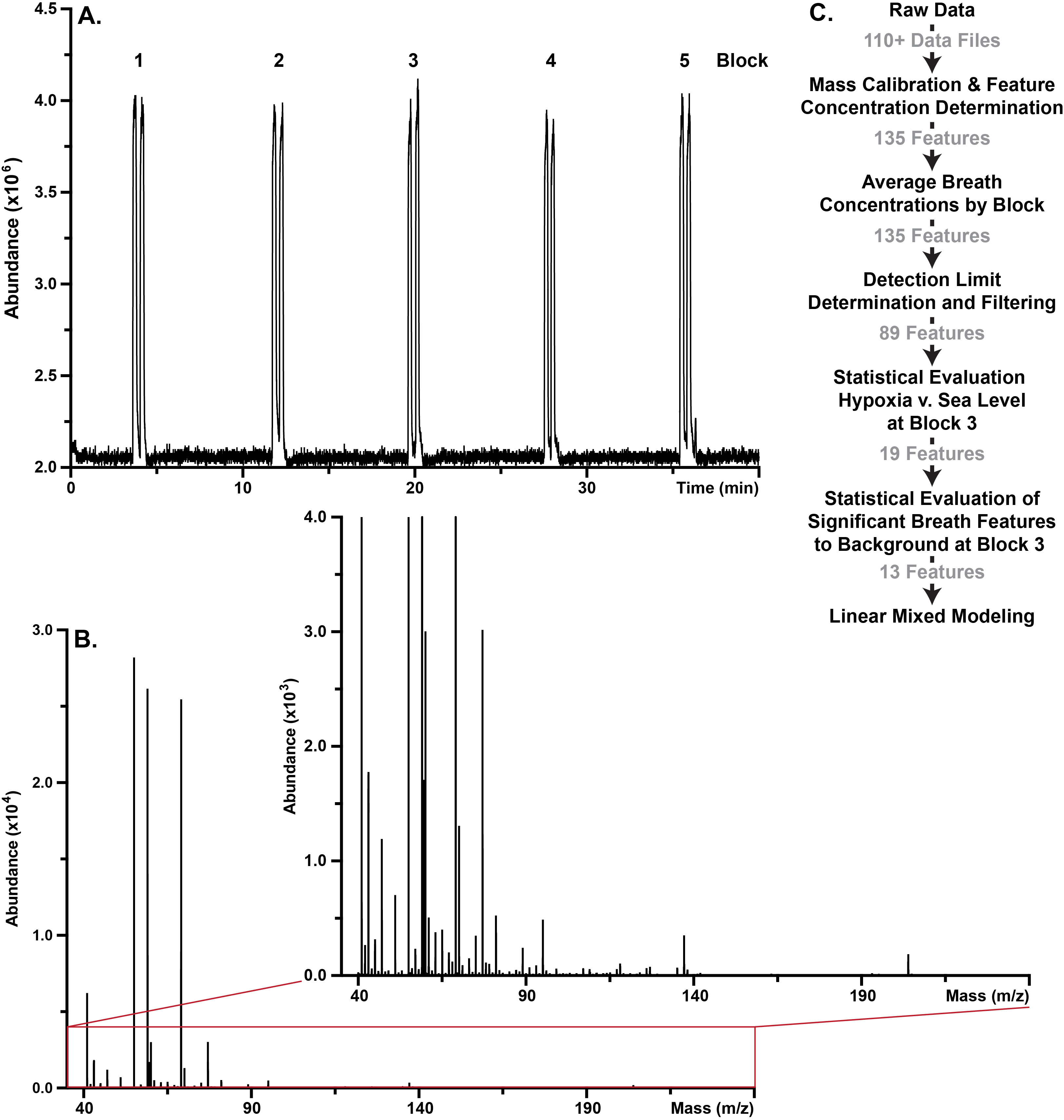


**SUPPLEMENTAL DATA 12:** **A)** A plot of the log_2_ concentration (ppb) of select exhaled breath features compared to background log_2_ concentration (ppb) from sea level (normoxia) exposures at block 3. **B)** A plot of the log_2_ concentration (ppb) of select exhaled breath features compared to background log_2_ concentration (ppb) from hypoxia exposures at block 3.


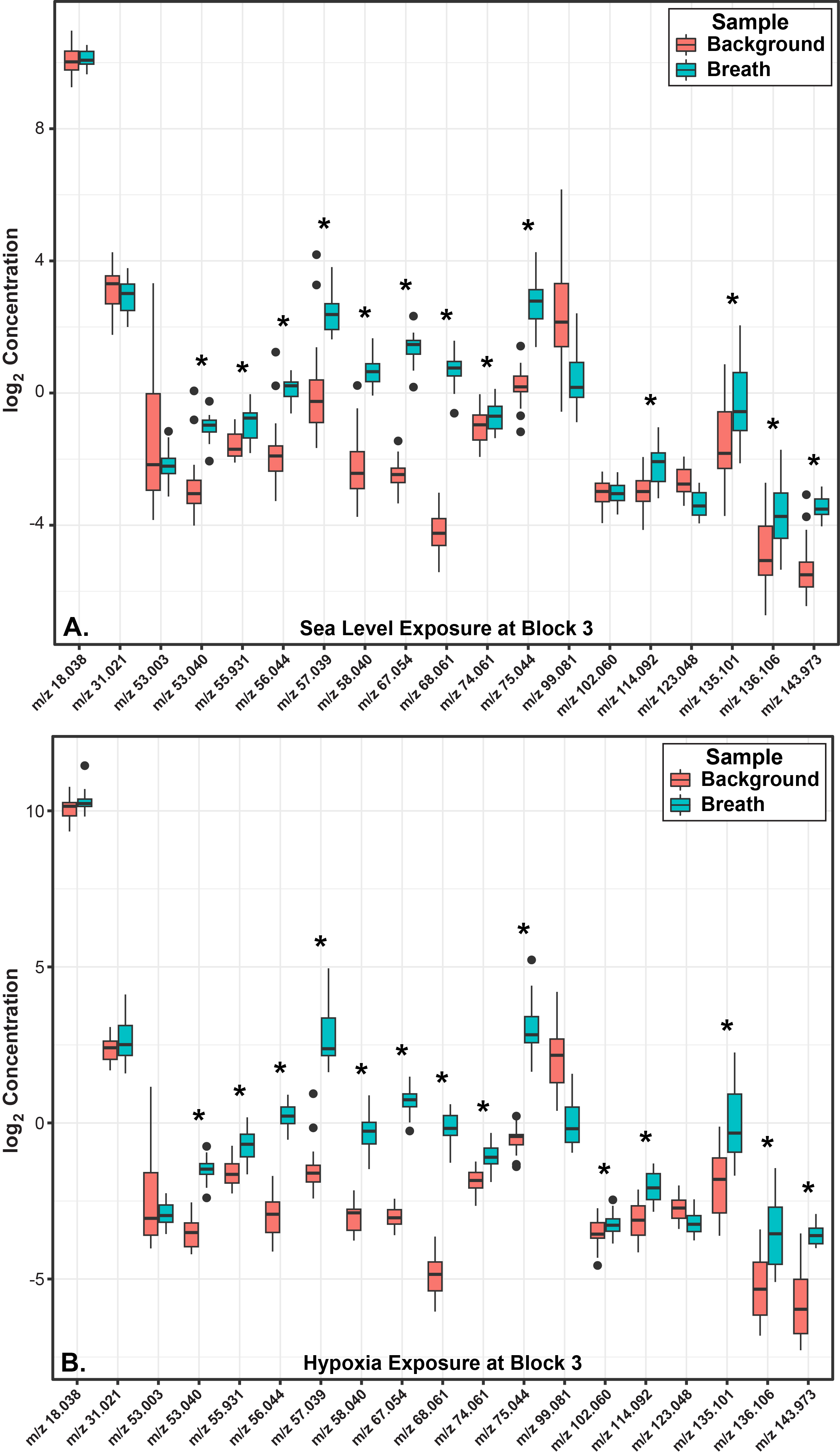

Supplement: Supplementary file 1 — Supplementary Material 1 [file 12931_2025_3296_MOESM1_ESM.docx]
